# Rehabilitation Robotics and Systems

**DOI:** 10.1155/2018/5370127

**Published:** 2018-12-17

**Authors:** Rafael Morales, José A. Somolinos, Antonio Fernández-Caballero, Carlo Ferraresi

**Affiliations:** ^1^Escuela de Ingenieros Industriales de Albacete, Universidad de Castilla-La Mancha, 02071 Albacete, Spain; ^2^Escuela de Ingenieros Navales, Universidad Politécnica de Madrid, Madrid, Spain; ^3^Politecnico di Torino, Torino, Italy

## 1. Introduction

Development of novel engineering solutions that incorporate robotic systems offers huge potential, as both assistive devices and therapeutic aids, for patients with reduced motor and/or cognitive abilities. Indeed, such technologies are capable of outperforming existing therapeutic systems for improving patients' achievable functional recovery. As a result, advanced engineering solutions for rehabilitation robotics have received a considerable amount of interest in recent years, from both the academic community and the industrial sector. Emphasis is typically focused on the improvement of sensor systems, control engineering, computer vision, robotic mechanics, human-machine interfaces, modelling and simulation, in-built intelligence, and informatics, to meet the broad range of challenges posed during patient rehabilitation.

This special issue is aimed at exhibiting the latest research achievements, findings, and ideas in the field of rehabilitation robotics and systems. Researchers were invited to contribute with their original research articles, as well as review articles, that summarize the most recent developments in the field of rehabilitation robotics and systems.

Potential topics included but were not limited to the following:

- Plasticity and motor-learning robotic devices
- Assistive and therapeutic robotic aids
- Upper and lower limb rehabilitation robotics
- Human-machine and brain-machine interfaces
- Mobile, wearable, and prosthetic robotic devices
- Sensors, intelligent sensors, and sensor networks
- Artificial intelligence-based rehabilitation systems
- Complex, nonlinear, and intelligent systems
- Vision, awareness, perception, and signal processing
- Sensor networks for precise motion control and visual servoing
- Adaptive control, robust control, and active disturbance rejection control
- Modelling and simulation for robotic rehabilitation systems
- Networked rehabilitation robotics
- Intelligent automation of rehabilitation robotics
- Informational monitoring, control, and data fusion for rehabilitation systems

## 2. The Papers

A total of 22 papers were submitted to this special issue. After peer review, finally 13 of them were accepted and published, covering a wide range of the topics proposed in the call for papers.

Three interesting papers are based on reviews of literature papers on rehabilitation robotics and systems. Firstly, a work by G. Carpino et al. includes a meta-analysis of robot-mediated lower limbs rehabilitation for stroke-affected patients; it is aimed at evaluating the effectiveness of the robotic approach using wearable robots or operational machines with respect to the conventional approach. The primary assessed outcome is the patient's ability to regain walking independence, whereas the secondary outcome is the average walking speed. Also, E. D. Oña et al. conduct a systematic literature review to identify the contribution of robotics to upper limb neurorehabilitation, highlighting its relationship with the rehabilitation cycle, and to clarify the prospective research directions in the development of more autonomous rehabilitation processes. In addition, a series of technical requirements that should be considered when designing and implementing autonomous robotic systems for rehabilitation are presented and discussed. Lastly, T. Eiammanussakul and V. Sangveraphunsiri review the training activities that were realized by rehabilitation robots in the literature to offer insights into developing a novel robot suitable for stroke rehabilitation. The control system of the lower limb rehabilitation robot in sitting position of the authors' previous work is discussed in detail to demonstrate the behavior of the robot while training a subject. A preliminary experiment is conducted on a healthy subject to show that the robot can perform active assistive exercises with various training activities and assist the subject to complete the training with a desired level of assistance.

Next, other six papers are more directed towards the experimental evaluation of rehabilitation mechanics. Thus, W. Liang and Y. Yu propose the so-called manipulability inclusive principle (MIP) to evaluate the assistive mechanism's assistive feasibility and assistive effect through the manipulability comparison between the assisted limb and slave-active-assistive mechanism. The optimization based on MIP can make the assistive mechanism realize better kinematical performance and assistance. Another paper by B. D. M. Chaparro-Rico et al. presents an experimental characterization of NURSE, a device for arm motion guidance. The laboratory setup and testing modes are presented. Two exercises for the upper limb are used to test the NURSE behavior. Trajectories and linear accelerations are tested when the device performs the exercises with and without load. Z. Ray and E. D. Engeberg face the problem of autonomously preventing grasped objects from slipping out of prosthetic hands for limb-absent people. Their paper explores a human-inspired grasp reflex controller for prosthetic hands to prevent slip of objects when they are rotated. This human-inspired grasped object slip prevention controller is evaluated with six different objects in benchtop tests and by 12 able-bodied subjects during realistic tasks of daily life. Moreover, in another article, M. Wu, M. R. Haque, and X. Shen present a new sit-to-stand (STS) control approach for powered lower limb prostheses, which can regulate the power delivery of the prosthetic knee joint to obtain natural STS motion like that displayed by healthy subjects. Mimicking the dynamic behavior of the knee in both phases of the STS, a unified control structure provides the desired control actions by combining an impedance function with a time-based ramp-up function. In addition, H. Guang et al. present an interactive upper limb rehabilitation robot with a parallel mechanism and an isometric screen embedded in the platform to display trajectories. In the dynamic modeling for impedance control, the effects of friction and inertia are reduced by introducing the principle of virtual work and derivative of the Jacobian matrix. To achieve the assist-as-needed impedance control for arbitrary trajectories, the strategy based on orthogonal deviations is proposed. Simulations and experiments were performed to validate the dynamic modeling and impedance control. Lastly, in a study by M. R. Afzal et al., a wearable system based on the reaction wheel is used to deliver light-touch-based balance biofeedback on the subject's back. The system can sense torso tilt and, using reaction wheels, generates light-touch. A group of 7 healthy young individuals performed balance tasks under 12 trial combinations based on two conditions each of standing stance and surface types and three of biofeedback device status.

A last group of four papers is directly focused demonstrating implementations for rehabilitation. A paper by J. Cantillo-Negrete et al. presents the implementation of a brain-computer interface system, coupled to a robotic hand orthosis, driven by hand motor imagery of healthy subjects and the paralyzed hand of stroke patients. A novel processing stage is designed using a bank of temporal filters, the common spatial patterns algorithm for feature extraction, and particle swarm optimization for feature selection. The system's performance shows that it has potential to be used for hand rehabilitation of stroke patients. The aim of an article by J. C. Castillo et al. is in the line of robotic therapies in which a robot can perform partially or autonomously a therapy session, endowing a social robot with the ability of assisting therapists in apraxia of speech rehabilitation exercises. The authors integrate computer vision and machine-learning techniques to detect the mouth pose of the user, and on top of that, our social robot performs autonomously the different steps of the therapy using multimodal interaction. Then, M. A. Padilla-Castañeda et al. introduce the development and evaluation of a robotic-assisted rehabilitation system as a new methodology of assisted physiotherapy in orthopedics. The proposal consists of an enhanced end-effector haptic interface mounted in a passive mechanism for allowing patients to perform upper limb exercising and integrates virtual reality games conceived explicitly for assisting the treatment of the forearm after injuries at the wrist or elbow joints. The authors design specific game scenarios enriched by proprioceptive and haptic force feedback in three training modes: passive, active, and assisted exercising. Finally, in a study by E. Beretta et al., a group of 18 children and adolescents with hemiplegia, an impairment due to acquired brain injuries, was enrolled and underwent intensive rehabilitation treatment including flank physical therapy and constrained-induced movement therapy or Armeo®Spring therapy. The effects of the treatments are assessed using clinical functional scales and upper limb kinematic analysis during horizontal and vertical motor tasks. The results of this study may be of help to define the best rehabilitation treatment for each patient, depending on the goal, and may thus support clinical decision.

